# Oral lactoferrin as a treatment of pediatrics’ anemia resulted from chronic kidney diseases: a randomized controlled trial

**DOI:** 10.1038/s41598-025-88506-2

**Published:** 2025-02-05

**Authors:** Sahar Kamal Hegazy, Mai Salah El-Din Koura, Mohamed Shokry Elharoun

**Affiliations:** 1 Professor of Clinical Pharmacy-Faculty of Pharmacy, Tanta University, Tanta, 31527 Egypt; 2 Senior Critical Care Clinical Pharmacy, Menoufia University Hospitals, MSc in pharmacy, Faculty of Pharmacy, Tanta University, Shebin El-Kom, 32511 Egypt; 3 Lecturer of Pediatrics-Faculty of Medicine, Menoufia University, Shebin El-Kom, 32511 Egypt

**Keywords:** Chronic kidney disease, Pediatrics, Anemia, Lactoferrin, IV iron dextran, Medical research, Nephrology

## Abstract

Anemia in pediatrics is often associated with chronic conditions such as chronic kidney disease (CKD). It can worsen the disease prognosis and affect quality of life. Injectable dosage forms are predominantly used in its treatment with various side effects. This randomized and parallel clinical trial aimed to compare the effectiveness of oral lactoferrin with intravenous (IV) iron dextran in managing anemia resulted from CKD in pediatrics. The study involved 60 children diagnosed with CKD-related anemia who were allocated into two separate groups. Group 1 consisted of 30 pediatric patients who received 100 mg of oral lactoferrin daily for a period of 3 months. Group 2 included 30 pediatric patients who were given IV iron dextran at a dosage of 50 mg three times weekly for 3 months. Both treatments are effective in treating CKD-induced anemia in pediatrics; however, oral lactoferrin demonstrated superior efficacy as there was a significant change within that group in levels of Hb, RBCs, MCH, iron, RDW-SD, MCHC, IL-6, and GDF-15 before and after treatment. In contrast, IV iron dextran showed significant changes within its group in iron, GFR, IL-6, GDF-15, and RDW-SD. After 3 months of treatment, no significant differences were observed between the two groups.

## Introduction

Chronic kidney disease (CKD) can be defined based on the estimated glomerular filtration rate (eGFR), where a diagnosis of CKD is made when the eGFR is consistently below 60 ml/min/1.73 mt2 for at least 3 months^1^.

CKD is a condition characterized by a gradual decline in kidney function. It can range from being asymptomatic if there is a structural issue in the kidney to severe impairment that significantly impacts the life expectancy and quality of life of patients. In some cases, CKD may necessitate the use of renal replacement therapy, such as dialysis or kidney transplantation^1^.

Anemia is a frequent complication among pediatrics with CKD^2^. It is one of the biological causes that affect CKD children’s quality of life, leading to reduced survival, energy, growth and academic performance^2,3^.

Risk factors for iron deficiency in CKD include blood loss, especially during dialysis or bleeding, reduced absorption of iron and inflammation^4^.

Anemia of chronic disorder is mainly hypochromic and microcytic anemia presented by low hemoglobin (Hb) values, low mean corpuscular volume (MCV) and mean corpuscular hemoglobin (MCH)^5^.

Hepcidin plays a defense role in inflammation. Interleukin-6 (IL-6) is one of the pro-inflammatory cytokines that induce the synthesis of hepcidin^6^.

High levels of IL-6 during inflammation lead to high levels of hepcidin expression. Increasing hepcidin levels can reduce oral iron absorption in patients with CKD. Another cause of increasing hepcidin levels is frequent doses of iron supplements. Decreased oral iron bioavailability results in a reduced response to these preparations and supports switching to IV iron preparations^7^.

Growth differentiation factor-15 (GDF-15) is thought to have a potential kidney protective effect. It can protect the kidney by down-regulating inflammatory markers and up-regulating anti-inflammatory markers. Increased GDF-15 levels are considered one of CKD risk factors^8^.

GDF-15 is an important regulator of hepcidin levels. There is a significant and positive link between these factors in people with anemia^9^.

As a result of gastrointestinal tract (GIT) adverse effects, the lack of adherence rate to oral iron preparations is highly significant among CKD pediatric patients^7^.

Lactoferrin (Lf) is a multifunctional iron-binding glycoprotein in mucosal secretions. It is an immune-modulator, antibacterial, antiviral and anti-inflammatory protein^10,11^.

Lactoferrin is an effective therapy for treating anemia in pediatrics and adults with almost no adverse effects^11^.

While oral iron therapy is commonly used and cost-effective for treating pediatric iron deficiency anemia (IBD), multiple studies have demonstrated that lactoferrin effectively improves hematological parameters, such as Hb and serum iron levels, more than oral iron preparations^11^.

The process by which lactoferrin treats anemia is believed to involve its strong binding to two iron ions, which leads to increased absorption of iron and enhanced uptake of iron into intestinal cells due to the presence of its own receptors. Lactoferrin releases iron within the intestinal cell, which is then transferred to the bloodstream through transferrin. Lactoferrin has the capacity to regulate inflammatory genes, which results in a decrease in the amount of IL-6. This decrease in IL-6 can reduce hepcidin and improve iron absorption and hemostasis^11,12^.

The choice of iron preparation dosage form is based on achieving desired outcomes, ensuring tolerance, maximizing convenience, and considering the response to previous therapies. Decreased intestinal iron absorption and increased gastrointestinal adverse effects cause reduced effectiveness of oral iron medications, leading to the necessity of using intravenous (IV) iron preparations for the treatment of anemia^13^.

Intravenous iron preparations are composed of colloids containing elemental iron enclosed by a carbohydrate shell. This shell serves to slow down the release of elemental iron, hence reducing the occurrence of severe adverse responses that might come from the sudden release of bioactive-free iron^13^.

Low doses of intravenous (IV) iron may be necessary to maintain hemoglobin levels and prevent anemia in CKD children after initial iron repletion therapy^14^, but with higher side effects.

The primary aim of this study was to compare between the effect of oral lactoferrin and IV iron dextran in treatment of CKD induced anemia. The second aim was to determine the efficacy and safety of oral lactoferrin in treatment of CKD induced anemia.

## Discussion

The objective of this study was to compare between the efficacy of different treatment options in treating pediatric CKD-induced anemia, we studied the efficacy of oral lactoferrin and IV dextran on CKD pediatric patients who were on regular dialysis or weren’t on dialysis.

The study was conducted as a randomized and parallel clinical trial which was done on 60 CKD pediatric patients.

The demographic data of all participants in both groups collected and mentioned in Table 1.

**Table 1:** The demographic data of the participants

Variable		Group 1	Group 2
		Mean ± SD	Mean ± SD
Age (Years)		10.52 ± 3.64	10.5 ± 3.5
Weight (Kg)		33.14 ± 14.20	27.3 ± 10.4
Height (cm)		118.17 ± 37.08	112.5 ± 41
BMI		27.76 ± 16.99	28.8 ± 19.5
Stage	4	8(26.7%)	0(0%)
	5	22(73.3%)	30(100%)

Data are presented as mean ± SD, BMI: Body mass index.

Group 1: 30 pediatric patients with CKD induced anemia administered oral lactoferrin 100 mg/day for 3 months.

Group 2: 30 pediatric patients with CKD induced anemia administered IV iron dextran 50 mg/3 times weekly for 3 month.

The results of parameters change throughout the treatment course which was 3 months to compare results after treatment between the two groups for each variable individually indicated no significant difference between two groups after 3 months of treatment in Hb (*p* = 0.592), RBCs (*p* = 0.706), WBCs (*p* = 0.670), MCH (*p* = 0.711), iron (*p* = 0.062), RDW-SD (*p* = 0.505), and MCHC (*p* = 0.321) levels. There was a significant difference between the two groups after 3 months of treatment in GDF-15 (*p* = 0.014) and TSAT (*p* = 0.004), IL-6 (*p* = 0.000) and GFR (*p* = 0.000) as mentioned in Table 2. Results indicate that both drugs had insignificant difference in their effect on CKD pediatric anemia after 3 months of treatment except the effect on IL-6, GDF-15, TSAT, and GFR. That indicates the ability of both drugs to treat CKD induced anemia.

**Table 2:** The change of the parameters of the participant before and after treatment

Variable	Group 1		Group 2		p-value
	Before	After	Before	After	
	Mean ± SD	Mean ± SD	Mean ± SD	Mean ± SD	
Hb (g/dl)	10.64 ± 1.38	11.37 ± 1.75	10.6 ± 1.6	11.2 ± 1.7	0.592
	P-value 0.006*		P-value 0.07		
RBCS (cellx10^6^/uL)	3.92 ± 0.69	4.18 ± 0.63	4 ± 0.7	4.1 ± 0.8	0.706
	P-value 0.000**		P-value 0.5		
WBCs (cellx10^3^/uL)	6.73 ± 2.39	6.58 ± 2.23	5.85 ± 1.62	6.52 ± 2.27	0.670
	P-value 0.584		P-value 0.146		
MCH (Pg)	27.22 ± 3	27.92 ± 3.29	27.83 ± 2.28	27.67 ± 2.47	0.711
	P-value 0.027*		P-value 0.674		
IL-6 (pg/mL)	150.19 ± 46.08	40.42 ± 11.83	77.43 ± 18.05	31.03 ± 13.03	0.000**
	P-value 0.000**		P-value 0.000**		
GDF-15 (pg/mL)	1659.61 ± 389.70	327.16 ± 177.67	906 ± 266.91	218.9 ± 124.39	0.002*
	P-value 0.000**		P-value 0.000**		
Iron (ug/dl)	76.50 ± 35.02	92.6 ± 34.36	63.8 ± 16.6	80.7 ± 42.5	0.062
	P-value 0.003*		P-value 0.014*		
TSAT (%)	38.01 ± 15.27	36.39 ± 10.64	27.02 ± 9.61	27.66 ± 12.01	0.004*
	P-value 0.773		P-value 0.746		
GFR (ml/min)	7.53 ± 2.73	6.73 ± 1.89	6.89 ± 2.37	6.17 ± 2.02	0.000**
	P-value 0.055		P-value 0.023*		
RDW-SD (fL)	44.81 ± 4.74	47.49 ± 4.97	45.11 ± 4.38	48.9 ± 5.49	0.505
	P-value 0.006*		P-value 0.000**		
MCHC (g/dl)	32.5 ± 1.65	33.09 ± 1.26	33.37 ± 1.65	33.43 ± 1.33	0.321
	P-value 0.049*		P-value 0.882		

Data are presented as mean ± SD**,** significant P-value < 0.05 (*) and highly significant P-value < 0.001 (**).

Hb: hemoglobin, RBCs: red blood cells, WBCs: white blood cells, MCH: mean corpuscular hemoglobin, IL-6: interleukin-6, GDF-15: growth differentiation factor-15, TSAT: transferrin saturation, GFR: glomerular filtration rate, RDW-SD: red cell distribution width—standard deviation, MCHC: Mean corpuscular hemoglobin concentration.

**Fig. 1:**
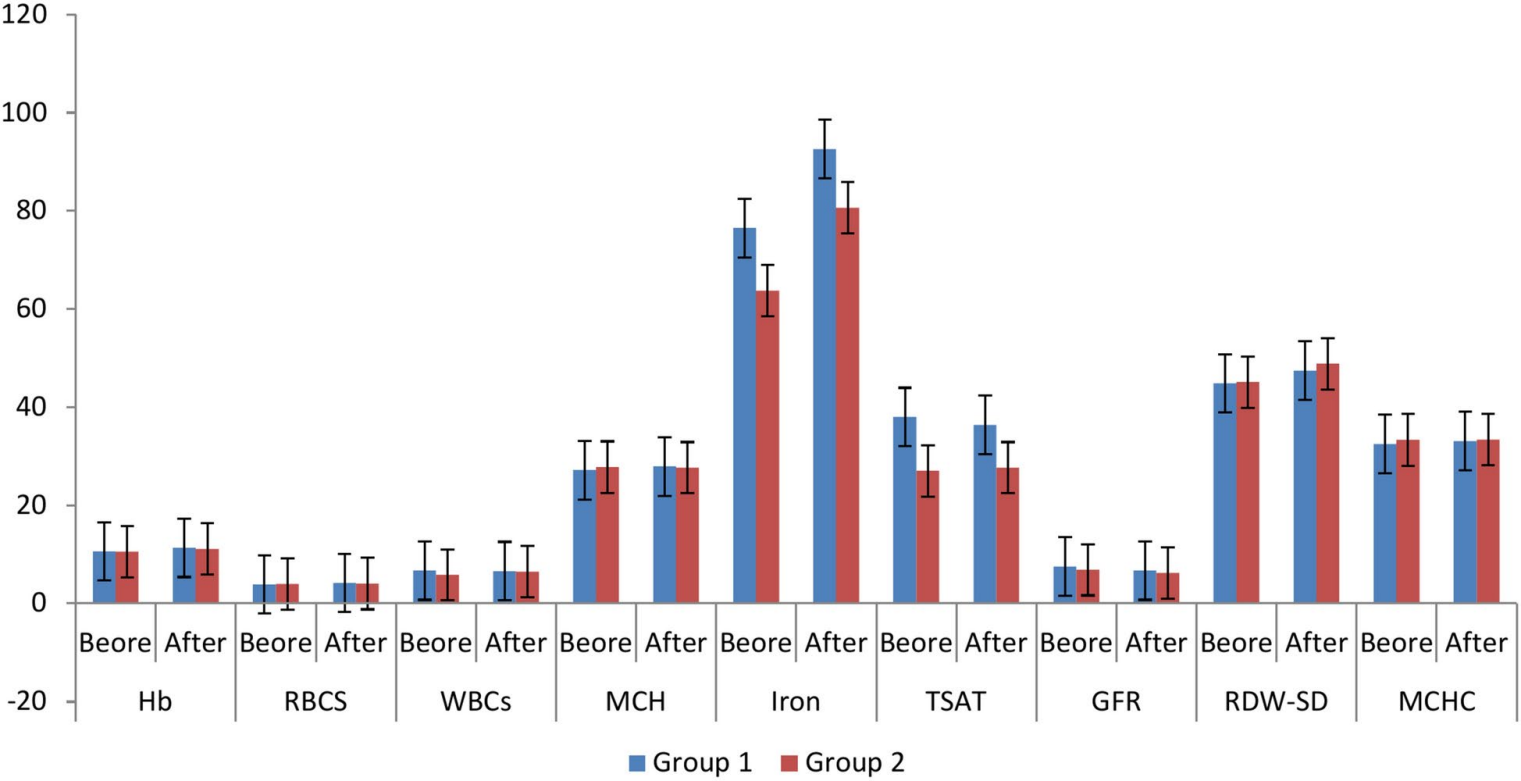
Results of Hb, RBCs, WBCs, MCH, TSAT, GFR, RDW-SD, and MCHC before and after treatment. Group 1: 30 pediatric patients with CKD induced anemia administered oral lactoferrin 100 mg/day for 3 months. Group 2: 30 pediatric patients with CKD induced anemia administered IV iron dextran 50 mg/3 times weekly for 3 month. Hb: hemoglobin, RBCs: red blood cells, WBCs: white blood cells, MCH: mean corpuscular hemoglobin, TSAT: transferrin saturation, GFR: glomerular filtration rate, RDW-SD: red cell distribution width—standard deviation, MCHC: Mean corpuscular hemoglobin concentration.

**Fig. 2:**
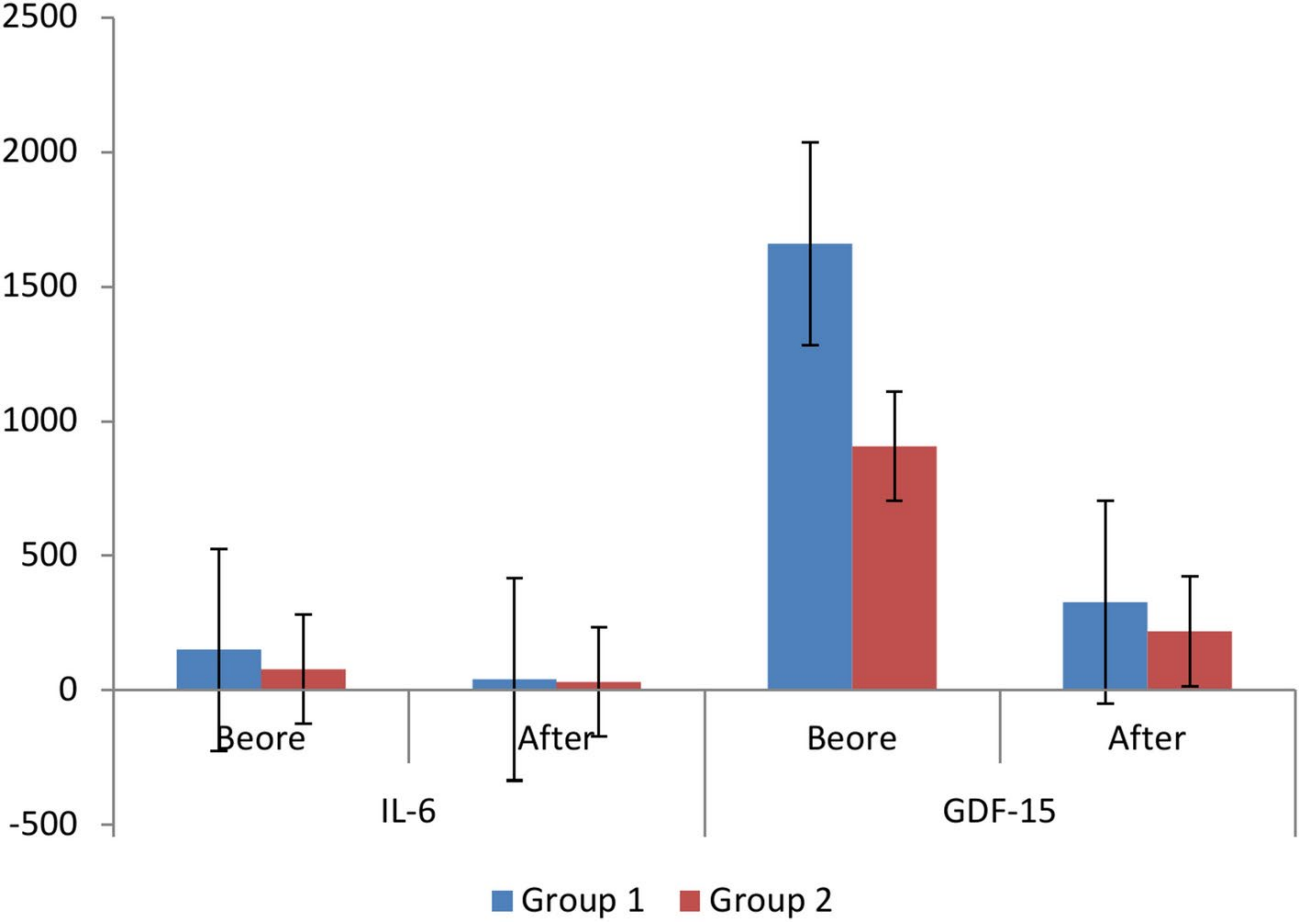
Results of IL-6 and GDF-15 before and after treatment. Group 1: 30 pediatric patients with CKD induced anemia administered oral lactoferrin 100 mg/day for 3 months. Group 2: 30 pediatric patients with CKD induced anemia administered IV iron dextran 50 mg/3 times weekly for 3 month. IL-6: interleukin-6, GDF-15: growth differentiation factor-15.

The results of parameters change throughout the treatment course which was 3 months in group 1 (lactoferrin group) to compare before and after treatment at the group for each variable individually indicated no significant difference between before and after treatment results in WBCs (*p* = 0.584), TSAT (*p* = 0.773), and GFR (*p* = 0.055) levels. Still, there was a significant difference between before and after treatment results in Hb (*p* = 0.006), MCH (*p* = 0.027), Iron (*p* = 0.003), RDW-SD (*p* = 0.006), and MCHC (*p* = 0.049), RBCS (*p* = 0.000), IL-6 (*p* = 0.000), and GDF-15 (*p* = 0.000) levels as mentioned in Table 2 and Figs. 1, 2.

Although, the results of parameters change throughout the treatment course which was 3 months in group 2 (IV dextran group) to compare before and after treatment at the group for each variable individually indicated no significant difference between before and after treatment results in Hb (*p* = 0.07), RBCs (*p* = 0.5), WBCs (*p* = 0.146), MCH (*p* = 0.674), TSAT (*p* = 0.746), and MCHC (*p* = 0.882) levels. There was a significant difference between before and after treatment results in iron (*p* = 0.014) and GFR (*p* = 0.023), IL-6 (*p* = 0.000), GDF-15 (*p* = 0.000), and RDW-SD (*p* = 0.000) as mentioned in Table 2.

These results indicate that lactoferrin has more effectiveness than IV dextran in treating CKD pediatric anemia, because lactoferrin caused significant differences within the group after 3 months of treatment in nearly all parameters except for only three parameters (WBCs, TSAT, and GFR). However, IV dextran caused significant differences within the group after 3 months of treatment in five parameters (iron, GFR, IL-6, GDF-15, and RDW-SD) and non-significant difference in the remaining six parameters.

Our results agree with the results of another clinical trial that was conducted on primary school children to treat IDA. Oral lactoferrin significantly increases RBC count, Hb concentration, serum ferritin and serum iron after one month of the treatment. The observed outcomes could be attributed to lactoferrin’s greater attraction to iron compared to serum transferrin^16^.

End results of many systematic reviews and meta-analysis comparing lactoferrin with ferrous sulfate has found that lactoferrin demonstrates a higher efficacy on serum iron, serum ferritin, hemoglobin concentration, circulating iron, and iron storage level with less gastrointestinal side effects^17,18^.

Lactoferrin can increase Hb concentration, iron absorption, and anti-inflammatory effects in CKD patients; these effects may be due to the increase in the erythroferrone hormone. This hormone can increase patient response to iron and decrease hepcidin^19^. Also, it can impact iron balance by promoting the export of iron from the gastrointestinal tract (GIT) and improving its storage in ferritin^16^.

Another suggested mechanism to illustrate lactoferrin’s effect on CKD-induced anemia is that lactoferrin has structural similarities like transferrin, has a greater affinity for binding to iron across a wide range of pH values, retains its ability to bind to iron even after being heated, and has the potential to increase the absorption of iron in the intestines and promote the formation of hemoglobin^16,18^.

In CKD, iron deficiency anemia patients have poor compliance to oral iron preparations; iron preparations can cause annoying gastrointestinal side effects. Due to lactoferrin enhanced safety and greater patient adherence, it can serve as an alternative treatment for patients experiencing negative side effects from oral iron therapy or can be added to oral iron treatment to enhance hemoglobin levels more effectively than iron alone^17,20^.

In a previous clinical study, lactoferrin had a significant decrease in serum hepcidin level and increase in Hb and TSAT compared to the ferrous glycine sulfate group^21^. Hepecidin is a hormone that is responsible for iron regulation in the body. Several causes can result in increased hepcidin in CKD patients, such as increased IL-6 levels, decreased renal metabolism and hepcidin excretion, increased tissue iron stores and increased inflammatory state. IL-6, hepcidin, and GDF-15 responsible for iron hemostasis and increasing of them lead to anemia and inflammation^9,22^ and ^23^. Lactoferrin has an anti-inflammatory effect, so it can reduce serum IL-6 levels, which is responsible for increasing hepcidin serum levels^18,21^. In our results both lactoferrin and IV dextran caused a highly significant decrease in IL-6 levels. The significant decrease in IL-6 can be an indicator in decreasing hepecidin levels that leads to better enhancement in serum iron levels and treatment of anemia resulted from CKD.

GDF-15 is an anti-inflammatory cytokine responsible for hepcidin regulation. Also, a high level of GDF15 may increase iron load in patients. So, any medication that decreases its concentration can decrease iron toxicity due to iron overload inside the body^24^. In our study both drugs caused a highly significant decrease in serum GDF-15 which indicates decreasing in inflammatory state in the human body and good regulation of iron.

It was noticed that the levels of serum GDF-15 are significantly higher in children with CKD, which may be due to GDF-15 having a negative feedback mechanism that suppresses elevated levels of hepcidin in patients with CKD and anemia may be attributed to the stimulation of GDF-15 production in red blood cells, which occurs as a consequence of iron deficiency in macrophages, these macrophages are activated with pro-inflammatory cytokines which increased in case of CKD^9^.

In IV dextran group GFR had a significant decrease after the treatment period, which may indicate deterioration in kidney functions; this may be due to oxidative stress produced by IV iron administration^25^.

IV dextran is effective in treatment of CKD induced anemia, but it has a lot of well-known side effects such as anaphylaxis, increased serum iron which may lead to iron toxicity, higher cost and higher risk of infection^26,27^. Also, it was noticed that IV iron dextran doesn’t produce increasing Hb levels; this may be due to introducing higher amounts of iron into the body that leads to decrease transferrin which is responsible for transporting iron to bone marrow for synthesis of new hemoglobin^28^. On the other hand lactoferrin proved to have higher efficacy with no mentioned side effects during the whole treatment period.

A potential limitation for our study is unavailability of other clinical trials that determined oral lactoferrin doses in anemia treatment so we used doses according to manufacture labels. Other longitudinal studies should be made with different doses to determine the most convenient dose in treatment of anemia.

Our study has a lot of strengths such as: (1) randomized, parallel, and controlled clinical trial which depended on simple randomization method (2) participated population had relative demographic parameters, and (3) introduced results that are helpful and applicable for CKD pediatrics.

## Conclusion

The summery of our study is that both drugs can be effective in treatment of CKD induced anemia but lactoferrin has greater efficacy with fewer side effects. Lactoferrin is a reasonable oral alternative to injectable preparations with more patient compliance and good prognosis in treating anemia resulted from CKD and enhancing underlying inflammatory state.

## Methods

### Study design

This is a randomized parallel study which was done at Menoufia University Hospitals in Pediatrics Nephrology Department and Pediatric Hemodialysis unit on 60 CKD pediatric patients. The 1964 Helsinki Declaration and its later revisions’ ethical guidelines were followed when conducting the study. The Menoufia University Research Ethics Committee approved the study, and it was registered on ClinicalTrials.gov under the NCT05714176, first trial registration date is 06/02/2023. All participants received information about the benefits and possible drawbacks of the research. Written informed consent was provided by each patient or their caregiver. The study was carried out between February 2023 and March 2024. Patients included in this study were children and adolescents less than 18 years old and CKD patients in stages 3–5. All pediatric patients in the study in both groups were on erythropoietin. Pediatric patients who were older in age, had bleeding, cancer or any other cause of anemia were excluded. The demographic and clinical characteristics of participated patients are included in the supplementary. In this study, we used the CONSORT reporting guidelines^29^.

### Study population and participants

**Fig. 3:**
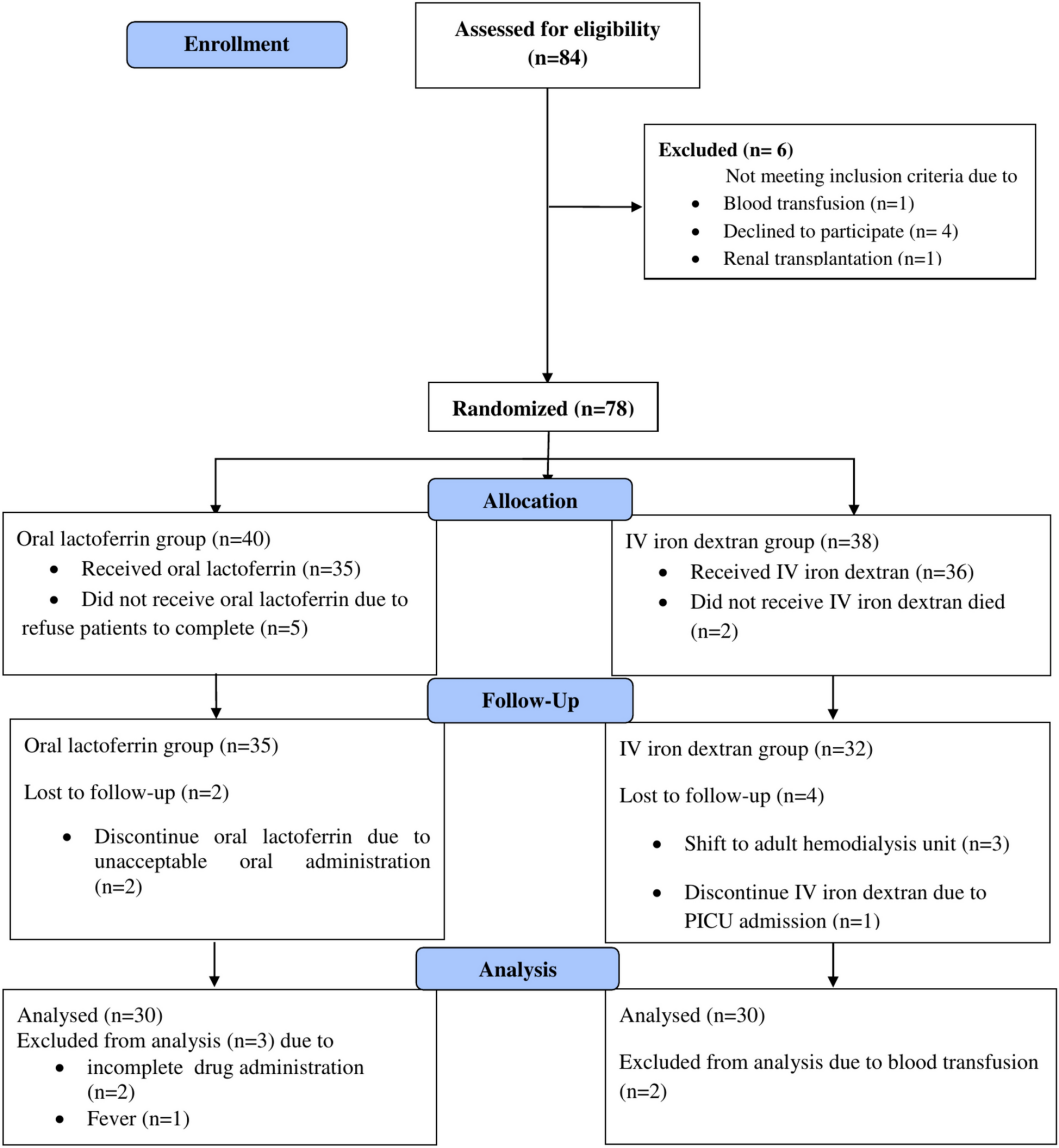
CONSORT flow diagram of the participants.

Patients were divided into 2 groups using simple randomization method as shown in CONSORT flow diagram (Fig. 3), group l: 30 pediatric patients who received oral bovine lactoferrin 100 mg/day for 3 months and group 2: 30 CKD pediatric patients who were on IV iron dextran 50 mg 3 doses in each week for 3 months.

### Sample size calculation

The sample size was calculated using an attrition rate of 10% on sample size of a previous follow-up cohort clinical study which included 45 CKD pediatric patients^15^. The resulted total sample size was 60 pediatric patients; each group consisted of 30 patients.

### Clinical end points

The main primary clinical endpoint was increasing in red blood cell count and hemoglobin level which would lead to improving in oxygen-carrying capacity of the blood. The secondary clinical endpoint was the change happened to all biological markers.

Laboratory measurements made before and after 3 months were Hb, RBCs, white blood cells (WBCs), MCH, red cell distribution width—standard deviation (RDW-SD), and Mean corpuscular hemoglobin concentration (MCHC). Biological biomarkers were IL-6, GDF-15, and Serum Iron. Calculated parameters were transferrin saturation (TSAT) and eGFR using Schwartz equation.

### Biochemical analysis

Blood samples from 60 CKD pediatric patients were collected before and after 3 months of treatment. Blood samples were divided into two parts. The first part used as complete blood sample to examine Hb, RBCs, WBCs, MCH, RDW-SD, and MCHC using Hematology Analyzer BC-20s Mindray® – Chinese using Mindray Bc20s Hematology Reagent Diluent, China. Serum Iron was measured calorimetry using ARCHITECT ci4100 (Abbott®-United States).

The second part was separated using a centrifuge operating at 4000 revolutions per second in a medical laboratory electric desktop 80–1 low-speed centrifuge (CNWTC®, China) to optain serum samples to examine IL-6 and GDF-15.

IL-6 and GDF-15 were measured with enzyme-linked immunosorbent assay (ELISA) using PR 4100 Absorbance Microplate Reader (BIO RAD®, USA). IL-6 analysis was done using ELISA kit Dldevelop company, China, Catalog Number DL-IL6-HU 96 Tests. GDF-15 analysis was done using ELISA kit Dldevelop company, China, Catalog Number DL-GDF15-Hu 96 Tests.

### Statistical analysis

Microsoft® Office Excel, 2019 (Microsoft Corporation) was used to organize and analyze data. IBM-SPSS statistical package version 26.0 (IBM corporation software group, USA) was used to conduct statistical analysis. Mean, standard deviation, and range were used to expressing numerical variables. A significance level P < 0.05 (*) determined a significant difference, although a significance level P < 0.001 (**) determined a highly significant difference.

The tests employed in this analysis:

- The normality test is used to examine the normality of the data.
- For parametric variables: unpaired t-test was used to compare the studied groups, and the Paired t-test was used to compare before and after treatment in each group.
- For nonparametric variables: Mann–Whitney test was used to compare the two groups and Wilcoxon test was used to compare between before and after treatment in each group.

## Data Availability

The datasets generated during and/or analysed during the current study are available from the corresponding author on reasonable request.

## References

[1] Vaidya, S. R., & Aeddula, N. R. Chronic kidney disease. *StatPearls - NCBI Bookshelf*. https://www.ncbi.nlm.nih.gov/books/NBK535404/ (2024). 30571025

[2] Carlson, J. et al. A longitudinal analysis of the effect of anemia on health-related quality of life in children with mild-to-moderate chronic kidney disease. *Pediatr. Nephrol.* **35**, 1659–1667 (2020). 32333284 10.1007/s00467-020-04569-5 PMC8958595

[3] Khalid, R. et al. Association between socioeconomic status and academic performance in children and adolescents with chronic kidney disease. *Pediatr. Nephrol.* **37**, 3195–3204 (2022). 35355084 10.1007/s00467-022-05515-3 PMC9587100

[4] Amanullah, F., Malik, A. A. & Zaidi, Z. Chronic kidney disease causes and outcomes in children: Perspective from a LMIC setting. *PLoS One* **17**(6), e0269632. 10.1371/journal.pone.0269632 (2022). 35675292 10.1371/journal.pone.0269632 PMC9176774

[5] Moscheo, C. et al. New insights into iron deficiency anemia in children: A practical review. *Metabolites.* **12**(4), 289 (2022). 35448476 10.3390/metabo12040289 PMC9029079

[6] Ganz, T. Iron biology: Metabolism and homeostasis. *Springer eBooks.* **19–33**, 2022. 10.1007/978-3-031-14521-6_2 (2022).

[7] Maladkar, M., Sankar, S. & Yadav, A. A novel approach for iron deficiency anaemia with liposomal iron: Concept to clinic. *JBM.* **8**(09), 27 (2020).

[8] Delrue, C., Speeckaert, R., Delanghe, J. R. & Speeckaert, M. M. Growth differentiation factor 15 (GDF-15) in kidney diseases. *Adv. Chem.* **114**, 1–46 (2023). 10.1016/bs.acc.2023.02.003 37268330

[9] Farag, N. M., Mousa, M., Elsayed, E. & Ismeil, A. GDF-15 and hepcidin as a therapeutic target for anemia in chronic kidney disease. *Ital. J. Pediatr.* **49**(1), 106 (2023). 37649102 10.1186/s13052-023-01505-9 PMC10469522

[10] Sienkiewicz, M., Jaśkiewicz, A., Tarasiuk, A. & Fichna, J. Lactoferrin: An overview of its main functions, immunomodulatory and antimicrobial role, and clinical significance. *Crit. Rev. Food Sci. Nutr.* **62**(22), 6016–6033 (2021). 33685299 10.1080/10408398.2021.1895063

[11] El Amrousy, D. et al. Lactoferrin for iron-deficiency anemia in children with inflammatory bowel disease: A clinical trial. *Pediatr. Res.* **92**, 762–766 (2022). 35681097 10.1038/s41390-022-02136-2 PMC9556315

[12] Zhao, et al. Comparative effects between oral lactoferrin and ferrous sulfate supplementation on iron-deficiency anemia: A comprehensive review and meta-analysis of clinical trials. *Nutrients.* **14**(3), 543. 10.3390/nu14030543 (2022). 35276902 10.3390/nu14030543 PMC8838920

[13] Gutiérrez, O. M. Treatment of iron deficiency anemia in CKD and end-stage kidney disease. *Kidney Int. Rep.* **6**(9), 2261–2269 (2021). 34514189 10.1016/j.ekir.2021.05.020 PMC8418942

[14] Ambarsari, C. G. et al. Low-dose maintenance intravenous iron therapy can prevent anemia in children with end-stage renal disease undergoing chronic hemodialysis. *Int. J. Nephrol.* **1**, 3067453. 10.1155/2020/3067453 (2020). 10.1155/2020/3067453 PMC7284959 32566294

[15] El-Farsy, M., El-Hakim, I. & Al-Arian, R. Role of oral lactoferrin as a source of iron supplementation in correction of anemia in pediatric patients with chronic kidney disease stages 2–4. *J. Egypt. Soc. Nephrol. Transplant.* **22**(4), 193–199 (2022).

[16] El-Khawaga, A. & Abdelmaksoud, H. Effect of lactoferrin supplementation on iron deficiency anemia in primary school children. *IJMA.* **1**(1), 48–52 (2019).

[17] Christofi, M. D. et al. The effectiveness of oral bovine lactoferrin compared to iron supplementation in patients with a low hemoglobin profile: A systematic review and meta-analysis of randomised clinical trials. *BMC Nutr.* **10**(1), 1–14 (2024). 38291525 10.1186/s40795-023-00818-6 PMC10825996

[18] Zhao, X. et al. Comparative effects between oral lactoferrin and ferrous sulfate supplementation on iron-deficiency anemia: A comprehensive review and meta-analysis of clinical trials. *Nutrients*10.3390/nu14030543 (2022). 35276902 10.3390/nu14030543 PMC8838920

[19] Kekan, K. et al. Wcn24-1274 the effect of lactoferrin on hemoglobin, erythroferrone and hepcidin levels in chronic kidney disease. *Kidney Int. Rep.* **9**(4), S119–S120 (2024).

[20] Divyaveer, S. et al. Wcn24-1323 to determine the efficacy of lactoferrin in improving hemoglobin levels in chronic kidney disease patients with iron deficiency anemia. *Kidney Int. Rep.* **9**(4), S120 (2024).

[21] Mahmoud, R. M. A. & Mohammed, A. lactoferrin: a promising new player in treatment of iron deficiency anemia in patients on regular hemodialysis: a randomized controlled trial. *Saudi J. Kidney Dis. Transplant.* **34**(3), 235–241 (2023). 10.4103/1319-2442.393996 38231718

[22] Sharkawy, M. M. E., Khedr, L. E., Abdelmbdy, A. H. & Mohamed, T. M. Role of hepcidin as a biomarker for iron status and its effect on anemia management in patients with chronic kidney disease (stage II-Iv) after HCV treatment. *QJM.*10.1093/qjmed/hcab100.128 (2021).

[23] Lee, N. H. Iron deficiency in children with a focus on inflammatory conditions. *Clin. Exp. Pediatr.* **67**(6), 283 (2024). 38772411 10.3345/cep.2023.00521 PMC11150987

[24] Youssry, I., Samy, R. M., AbdelMohsen, M. & Salama, N. M. The association between growth differentiation factor-15, erythroferrone, and iron status in thalassemic patients. *Pediatr. Res.* **95**(4), 1095–1100 (2024). 37464096 10.1038/s41390-023-02729-5 PMC10920194

[25] Rameshkumar, S., Viswanathan, S., Jagadeesan, A. R. & Dhanunjaya, Y. A pilot study on effect of intravenous iron sucrose on oxidative stress and antioxidant status of pregnant women with iron deficiency anemia. *Indian J. Med. Sci.*10.25259/IJMS_402_2021 (2023).

[26] Bazeley, J. W. & Wish, J. B. Recent and emerging therapies for iron deficiency in anemia of CKD: A review. *AJKD.* **79**(6), 868–876 (2022). 34758368 10.1053/j.ajkd.2021.09.017

[27] Ganz, T. et al. Iron administration, infection, and anemia management in ckd: untangling the effects of intravenous iron therapy on immunity and infection risk. *Kidney Med.* **2**(3), 341–353 (2020). 32734254 10.1016/j.xkme.2020.01.006 PMC7380433

[28] Ogun, A. & Adeyinka, S. A. *Biochemistry Transferrin* (StatPearls, Treasure Island (FL), 2025). 30422523

[29] Schulz, K. F., Altman, D. G. & Moher, D. WITHDRAWN: CONSORT 2010 Statement: Updated guidelines for reporting parallel group randomised trials. *Int. J. Surg.*10.1016/j.ijsu.2010.09.006 (2010). 10.1016/j.ijsu.2011.09.004 22019563

